# Plk1 regulates MEK1/2 and proliferation in airway smooth muscle cells

**DOI:** 10.1186/s12931-015-0257-8

**Published:** 2015-08-05

**Authors:** Sixin Jiang, Dale D. Tang

**Affiliations:** The Center for Cardiovascular Sciences, Albany Medical College, 47 New Scotland Avenue, MC-8, Albany, NY 12208 USA

## Abstract

**Background:**

Polo-like kinase 1 (Plk1) is a serine/threonine protein kinase that has been implicated in the regulation of mitosis. In addition, the activation of mitogen-activated protein kinase (MAPK) is a key event in the early stage of the growth factor response. The role of Plk1 in MAPK phosphorylation in cells has not been investigated.

**Methods:**

Immunoblot analysis was used to evaluate Plk1 and MAPK phosphorylation in cells upon stimulation with platelet-derived growth factor (PDGF). We also generated stable Plk1 knockdown (KD) cells to assess the role of Plk1 in MAPK activation and cell proliferation. Furthermore, we used a non-phosphorylatable Plk1 mutant to determine the function of Plk1 phosphorylation in these processes.

**Results:**

Treatment with PDGF increased Plk1 phosphorylation at Thr-210 (an indication of Plk1 activation) in human airway smooth muscle cells. Plk1 KD attenuated the PDGF-induced phosphorylation of MEK1/2 and ERK1/2 as well as cell proliferation. However, phosphorylation of Raf-1 and AKT upon stimulation with PDGF was not reduced in Plk1 KD cells. Furthermore, the expression of T210A Plk1 (alanine substitution at Thr-210) inhibited the PDGF-stimulated MEK1/2 phosphorylation, ERK1/2 phosphorylation and cell proliferation.

**Conclusions:**

Together, these findings suggest that Plk1 is activated upon growth factor stimulation, which may control the activation of MEK1/2 and ERK1/2, and smooth muscle cell proliferation.

## Introduction

Airway smooth muscle cell proliferation contributes to the pathogenesis of airway remodeling, a key characteristic of chronic asthma [1]. However, the mechanisms that regulate smooth muscle cell proliferation are not fully understood.

Polo-like kinase 1 (Plk1) is a serine/threonine protein kinase that has been implicated in cell-cycle-associated processes such as centrosome maturation, mitotic spindle assembly, sister chromatid cohesion and cytokinesis [2, 3]. Knockdown (KD) or depletion of Plk1 induces mitotic arrest in various nonmuscle cell types [2, 4, 5]. During mitosis and cytokinesis, the functions of Plk1 are regulated by its expression, spatial localization, and activation [2, 5].

The activation of Plk1 is largely regulated by phosphorylation at Thr-210 located in the catalytic domain of the kinase [2, 6]. *In vitro* biochemical studies suggest that phosphorylation at Thr-210 increases Plk1 activity [6, 7]. In addition, Plk1 is first activated in G2 and reaches maximal activity in mitosis, coincident with the kinetics of Thr-210 phosphorylation [4]. By phosphorylating distinctive substrates, activated Plk1 regulates centrosomal maturation, spindle organization and cytokinesis [2, 3].

The mitogen-activated protein kinase (MAPK) pathway plays an essential role in regulating various cellular functions including cell proliferation [8–10]. In response to stimulation with growth factors within minutes, MEK1/2 (MAPK kinase) gets phosphorylated by Raf-1 [10, 11], which in turn phosphorylates and activates ERK1/2. Activated ERK1/2 phosphorylates several protein kinases, transcription factors, and other proteins to promote cell proliferation eventually [8–11].

In addition to mitosis and cytokinesis, Plk1 has been implicated in the DNA damage response, development [3], cancer cell invasion [12], apoptosis and autophagy [13]. However, the role of Plk1 in the early stage of the growth factor response has not been investigated.

Here, we find that stimulation with platelet-derived growth factor (PDGF) induces Plk1 phosphorylation at Thr-210. KD of Plk1 inhibits the PDGF-induced phosphorylation of MEK1/2, ERK1/2 and smooth muscle cell proliferation. Furthermore, phosphorylation at Thr-210 is required for the Plk1-mediated activation of MEK1/2 and ERK1/2. Thus, we propose that Plk1 is a critical molecule that regulates the activation of MEK1/2 in smooth muscle cells during the cellular responses to growth factor stimulation.

## Materials and methods

### Cell culture

Human airway smooth muscle (HASM) cells were prepared from human bronchi and adjacent tracheas obtained from the International Institute for Advanced Medicine [8, 14–16]. Human tissues were non-transplantable and consented for research. This study was approved by the Albany Medical College Committee on Research Involving Human Subjects. Briefly, muscle tissues were incubated for 20 min with dissociation solution [130 mM NaCl, 5 mM KCl, 1.0 mM CaCl_2_, 1.0 mM MgCl_2_, 10 mM Hepes, 0.25 mM EDTA, 10 mM D-glucose, 10 mM taurine, pH 7, 4.5 mg collagenase (type I), 10 mg papain (type IV), 1 mg/ml BSA and 1 mM dithiothreitol]. All enzymes were purchased from Sigma-Aldrich. The tissues were then washed with Hepes-buffered saline solution (composition in mM: 10 Hepes, 130 NaCl, 5 KCl, 10 glucose, 1 CaCl_2_, 1 MgCl_2_, 0.25 EDTA, 10 taurine, pH 7). The cell suspension was mixed with Ham’s F12 medium supplemented with 10 % (v/v) fetal bovine serum (FBS) and antibiotics (100 units/ml penicillin, 100 μg/ml streptomycin). Cells were cultured at 37 °C in the presence of 5 % CO_2_ in the same medium. The medium was changed every 3–4 days until cells reached confluence, and confluent cells were passaged with trypsin/EDTA solution [8, 17, 18]. HASM cells (passage 3–10) from four non-asthmatic donors were used for experiments. They were serum starved for 24 h before treatment with PDGF.

### Immunoblot analysis

Cells were lysed with RIPA 150 (50 mM Tris–HCl, pH7.6, 150 mM NaCl, 2 mM sodium pyrophosphoate, 0.5 % sodium dexoycholate,1 % NP-40, 0.1 % SDS, 5 mM EDTA) with protease and phosphatase inhibitors (1 mM benzamidine, 1 mM PMSF, 1 μg/ml aprotinin, 1 μg/ml leupeptin, 1 μg/ml pepstatin, 2 mM sodium orthovanadate, 5 mM sodium fluoride). Cell lysates were mixed with SDS sample buffer composed of 1.5 % dithiothreitol, 2 % SDS, 80 mM Tris–HCl (pH 6.8), 10 % glycerol and 0.01 % bromophenol blue, and were boiled for 5 min and separated by SDS-PAGE. Proteins were transferred to a nitrocellulose membrane. The membrane was blocked with bovine serum albumin or milk for 1 h and probed with use of primary antibody followed by horseradish peroxidase-conjugated secondary antibody (Thermo Fisher Scientific). Proteins were visualized by enhanced chemiluminescence (Thermo Fisher Scientific) using the LAS-4000 Fuji Image System. Antibodies used were anti-phospho-Plk1 (Abcam), anti-Plk1 (EMD Millipore), anti-phospho-MEK1/2 (Santa Cruz Biotech.), anti-MEK1/2 (Santa Cruz Biotech.), anti-phospho-ERK1/2 (Cell signaling), anti-ERK1/2 (Cell signaling), anti-phospho-Raf-1 (Santa Cruz Biotech.), anti-Raf-1 (Santa Cruz Biotech.) and anti-GAPDH (Ambion). The levels of total protein or phosphoprotein were quantified by scanning densitometry of immunoblots (Fuji Multigauge Software). The luminescent signals from all immunoblots were within the linear range [14, 19–21].

### Assessment of cell proliferation

Cells were seeded in 24-well plates with the F12 medium with 10 % FBS for at least 18 h. Cells were then serum starved for 24 h. They were subsequently treated with PDGF-BB (20 ng/ml) in the medium containing 0.25 % FBS. Numbers of viable cells were counted using the trypan blue exclusion test [9]. Briefly, cell suspension was incubated with 0.4 % trypan blue for 5 min at room temperature. Unstained cells (viable cells) were then counted.

The BrdU (5′-bromo-2′-deoxyuridine) cell proliferation assay kit (Millipore) was also used to evaluate DNA synthesis. BrdU is an analog of thymidine, which is able to incorporate into newly-synthesized DNA. Cells in 96-well (1.2x 10^4^ cells/well) were treated with BrdU for 24 hrs. They were then fixed and reacted with BrdU antibody for 1 h, followed by incubation with secondary antibody conjugated with peroxidase. They were reacted with peroxidase substrates, and the reaction was detected using a Promega GloMax-Multi Microplate reader.

### Generation of stable Plk1 KD cells

We used lentivirus-mediated shRNA [14–16, 20] to generate stable Plk1 knockdown (KD) cells. Briefly, lentiviral particles encoding Plk1 shRNA (sc-36277-V) or control shRNA (sc-108080) were purchased from Santa Cruz Biotechnology. HASM cells were infected with control shRNA lentiviruses or Plk1 shRNA lentiviruses for 12 h. They were then cultured for 3–4 days. Positive clones expressing shRNAs were selected by puromycin. Immunoblot analysis was used to determine the protein levels of Plk1 in these cells. Plk1 KD cells and cells expressing control shRNA were stable at least five passages after initial infection.

### *In vitro* kinase assay

Active Plk1 (40 ng, Millipore) and 2 μg MEK1 (unactive, Millipore) were placed in 20 μl kinase buffer containing 20 mM HEPES (pH7.5), 60 mM NaCl, 2 mM MgCl_2_, 5 mM EGTA and 100 μM ATP. Kinase reaction mix was incubated at 30 °C for 30 min, and stopped by the addition of the SDS sample buffer [18, 22]. The samples were boiled for 5 min and separated by SDS-PAGE followed by membrane transfer. The membrane was probed with phospho-MEK1/2 antibody, stripped, and reprobed with MEK1/2 antibody.

### Mutagenesis, plasmid purification and cell transfection

T210A Plk1 (alanine substitution at Thr-210) was generated by using the Quick change II XL site-directed mutagenesis kit (Agilent Technologies). The template plasmid pcDNA3 3xFlag-Plk1 was kindly provided by Dr. David Stern (Yale University). The primers were designed using the online service PrimerX (http://www.bioinformatics.org/primerx/index.htm) and were synthesized by Invitrogen. The 5′ primer was 5′ –GGGGAGAGGAAGAAGGCCCTGTGTGGGACTC- 3′. The 3′ primer was 5′- GAGTCCCACACAGGGCCTTCTTCCTCTCCCC- 3′. The PCR product was digested with Dpn I at 37 °C for 2-3 h to remove the parental DNA, and was transformed into XL10- Gold Ultracompetenet cells (Agilent Technologies). Plasmids were purified by using the Pureklink Quick Plasmid Miniprep kit (Invitrogen). DNA sequencing was performed by Genewiz Life Sciences.

### Statistical analysis

All statistical analysis was performed using Prism 6 software (GraphPad Software, San Diego, CA). Comparison among multiple groups was performed by one-way analysis of variance followed by Tukey’s multiple comparison test. Differences between pairs of groups were analyzed by Student-Newman-Keuls test or Dunn’s method. Values of n refer to the number of experiments used to obtain each value. *P* < 0.05 was considered to be significant.

## Results

### PDGF stimulation induces phosphorylation of Plk1, MEK1/2 and ERK1/2 in smooth muscle cells

**Fig. 1:**
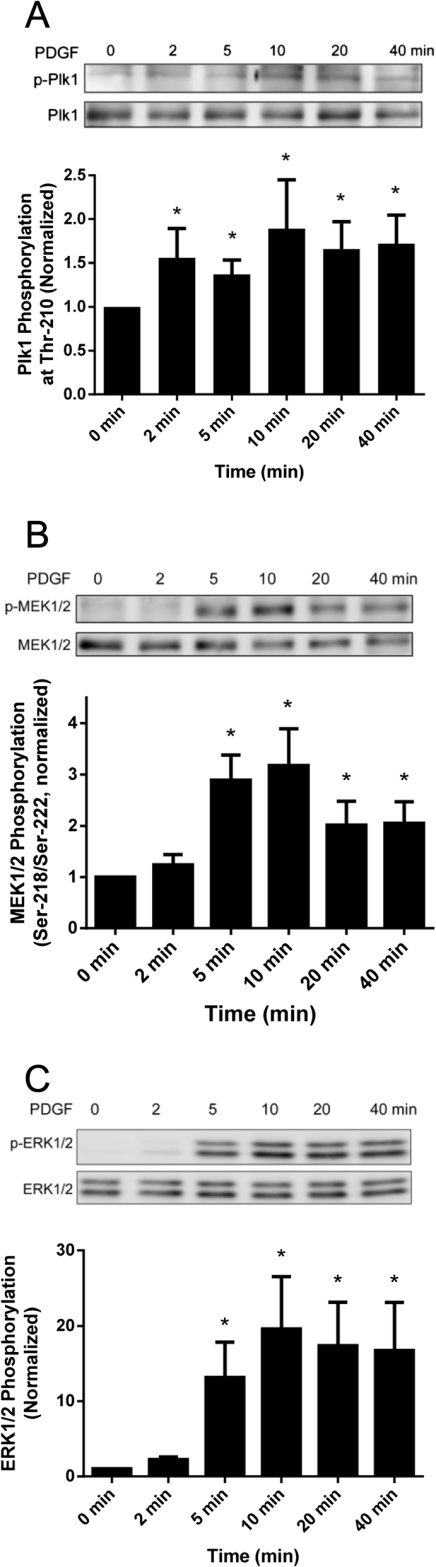
Stimulation with PDGF induces the phosphorylation of Plk1, MEK1/2 and ERK1/2 in smooth muscle cells. Human airway smooth muscle (HASM) cells were stimulated with 20 ng/ml PDGF for different durations or left unstimulated. The phosphorylation of Plk1 (**a**), MEK1/2 (**b**) and ERK1/2 (**c**) in these cells was evaluated by immunoblot analysis. The phosphorylation levels of the proteins in stimulated cells were normalized to corresponding unstimulated cells. * Significantly higher phosphorylation levels in stimulated cells compared with unstimulated cells (*P* < 0.05). Values represent mean ± SE (*n* = 4)

As described earlier, Plk1 has been implicated in mitosis and cytokinesis in nonmuscle cells [2, 4, 5]. The role of Plk1 in the early phase of the cellular responses to growth factor stimulation is largely unknown. We evaluated the effects of PDGF stimulation on Plk1 phosphorylation at Thr-210 (an indication of Plk1 activation) [6]. Human airway smooth muscle cells were serum starved for 24 h, which synchronizes the cells in G_0_-G_1_ [9, 20]. Cells were then stimulated with 20 ng/ml for different time periods, or left unstimulated. Plk1 phosphorylation at Thr-210 in the cells was assessed by immunoblot analysis. In unstimulated cells, the level of Plk1 phosphorylation at Thr-210 was relatively low. Stimulation with PDGF increased Plk1 phosphorylation, which was evident as early as 2 min after PDGF stimulation, and persisted for the 40-min duration of the stimulation (Fig. 1a).

Because the MAPK pathway has been implicated in growth factor-mediated signaling [8–10], we also determined the phosphorylation of MEK1/2 and ERK1/2 in smooth muscle cells. The phosphorylation of MEK1/2 and ERK1/2 was significantly increased 5 min after PDGF stimulation and maintained 40 min after the stimulation with PDGF (Fig. 1b and c).

### KD of Plk1 attenuates the phosphorylation of MEK1/2 and ERK1/2 upon PDGF stimulation

**Fig. 2:**
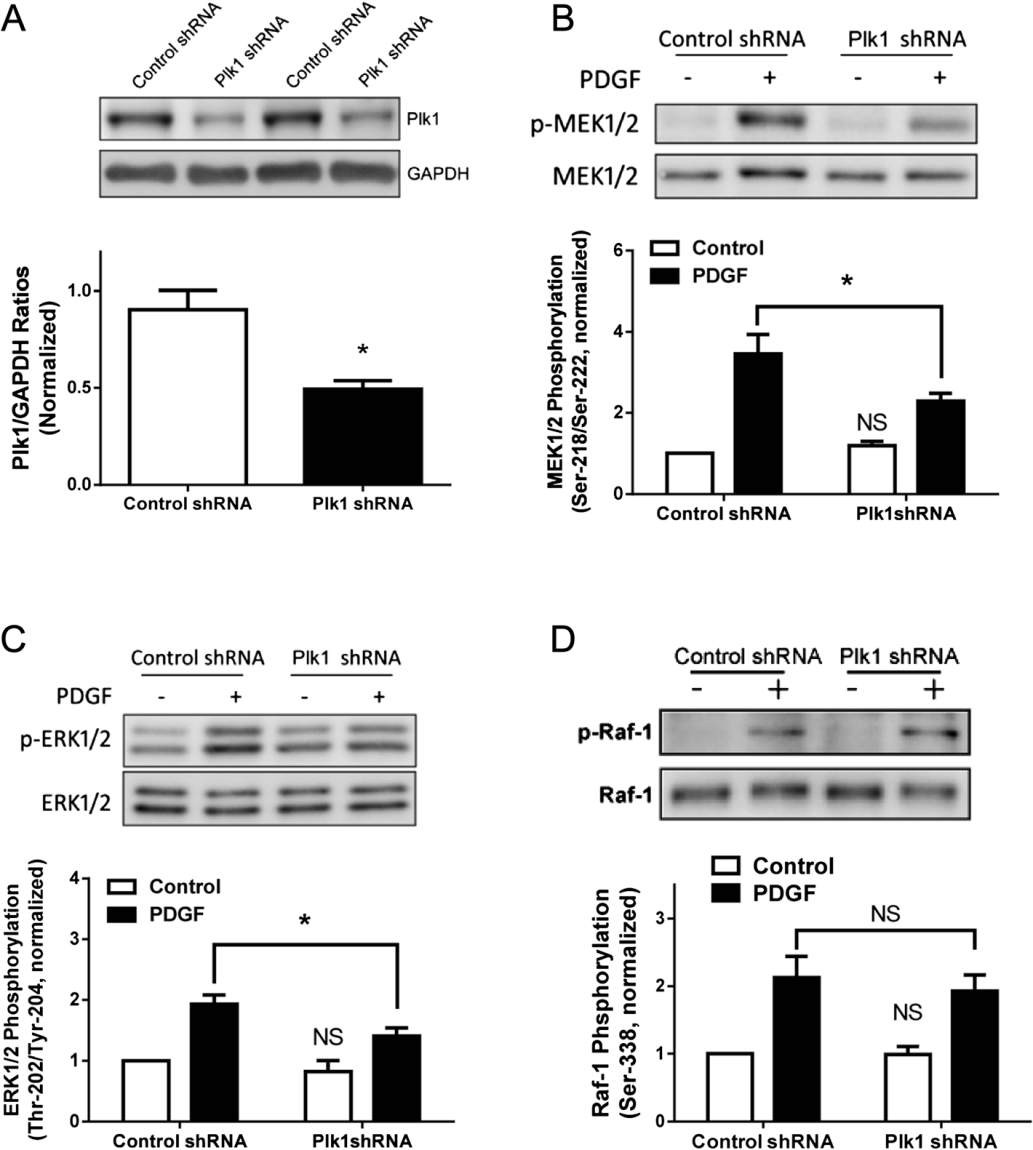
Knockdown (KD) of Plk1 attenuates the PDGF-induced phosphorylation of MEK1/2 and ERK1/2, but not Raf-1 phosphorylation. **a** Representative immunoblots illustrating the effects of Plk1shRNA on Plk1 expression. Blots of HASM cells infected with lentiviruses encoding control shRNA or Plk1 shRNA were probed with antibodies against Plk1 and GAPDH. Duplicated samples of each treatment are shown. Ratios of Plk1/GAPDH protein in cells producing Plk1 shRNA were normalized to ratios obtained from cells producing control shRNA. Values are mean ± SE (*n* = 4). *Significantly lower Plk1/GAPDH ratios in cells producing Plk1 shRNA compared with cells producing control shRNA (*P* < 0.05). **b** Cells expressing control shRNA or Plk1 shRNA were treated with 20 ng/ml PDGF for 10 min, or they were not stimulated. Protein phosphorylation was then evaluated by immunoblotting. MEK1/2 phosphorylation induced by PDGF was significantly reduced in Plk1KD cells compared to cells treated with control shRNA (*P < 0.05). NS, basal MEK1/2 phosphorylation in Plk1 KD was not significantly different from cells expressing control shRNA (*P* > 0.05). Values are mean ± SE (*n* = 8). **c** PDGF-induced ERK1/2 phosphorylation was significantly reduced in Plk1 KD cells (*P < 0.05, *n* = 7). NS, basal ERK1/2 phosphorylation in Plk1 KD was not significantly different from cells expressing control shRNA (*P* > 0.05). **d** PDGF-stimulated Raf-1 phosphorylation at Ser-338 was comparable in Plk1 KD cells and cells expressing control shRNA. NS, Raf-1 phosphorylation in Plk1 KD was not significantly different from cells expressing control shRNA (*P* > 0.05, *n* = 8). Protein phosphorylation under various treatments was normalized to the levels in unstimulated cells expressing control shRNA

To reveal the functional role of Plk1 in the growth factor-associated process, we evaluated the effects of Plk1 KD on MEK/1/2 and ERK1/2 phosphorylation in smooth muscle cells. Stable Plk1 KD cells were generated by using lentivirus-mediated shRNA. Immunoblot analysis showed that the protein level of Plk1 in cells infected with viruses for Plk1 shRNA was lower compared to control cells. However, the protein level of GAPDH was similar in these cells. Ratios of Plk1/GAPDH were lower in Plk1 KD cells than in control cells (Fig. 2a).

Plk1 KD cells and cells expressing control shRNA were stimulated with 20 ng/ml PDGF for 10 min or left unstimulated. The phosphorylation of MEK1/2 and ERK1/2 in the cells was determined by immunoblot analysis. In smooth muscle cells expressing control shRNA, the phosphorylation of MEK1/2 and ERK1/2 was increased in response to stimulation with PDGF. However, the PDGF-induced phosphorylation of MEK1/2 and ERK1/2 was reduced in Plk1 KD smooth muscle cells (Fig. 2b and c). Basal phosphorylation of MEK1/2 and ERK1/2 was not affected by Plk1 knockdown.

### Raf-1 phosphorylation is not reduced in Plk1 KD cells

Because Raf-1 is known to regulate MEK1/2 phosphorylation, we also evaluated the effects of Plk1 KD on Raf-1 phosphorylation in smooth muscle cells. Blots of cells treated with 20 ng/ml PDGF for 10 min were probed with antibodies against phospho-Raf-1 and total Raf-1. Raf-1 phosphorylation upon PDGF stimulation was similar in cells expressing control shRNA and Plk1 KD cells (Fig. 2d). Basal Raf-1 phosphorylation was also comparable in cells expressing control shRNA and Plk1 KD cells.

### Plk1 catalyzes MEK phosphorylation *in Vitro*

**Fig. 3:**
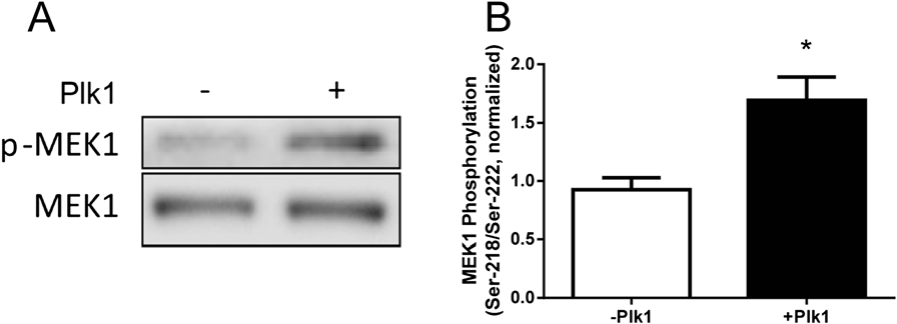
Plk1 catalyzes MEK phosphorylation *in vitro*. Purified active Plk1 (40 ng) and 2 μg purified unactive MEK1 were placed in kinase buffer. MEK phosphorylation was determined by immunoblot analysis 30 min after the initiation of the reaction. **a** Representative immunoblots illustrates Plk1-mediated MEK phosphorylation. **b** MEK phosphorylation catalyzed by Plk1 was normalized to the level of MEK phosphorylation in the absence of Plk1 (*n* = 4, *P < 0.05)

As described above, KD of Plk1 mediates the phosphorylation of MEK1/2 and ERK1/2 without affecting Raf-1 phosphorylation. Because ERK1/2 is phosphorylated by the dual-specificity protein kinase MEK1/2, and Plk1 is not a dual-specificity protein kinase [8, 10], it is unlikely that Plk1 catalyzes ERK1/2 directly. To verify whether Plk1 directly mediates MEK phosphorylation, purified active Plk1 was added to purified unactive MEK1 in kinase buffer. MEK phosphorylation was determined by immunoblot analysis. The addition of Plk1 to the kinase buffer containing MEK1 increased the phosphorylation of MEK (Fig. 3). The results suggest that Plk1 directly catalyzes MEK phosphorylation.

### KD of Plk1 inhibits smooth muscle cell proliferation

**Fig. 4:**
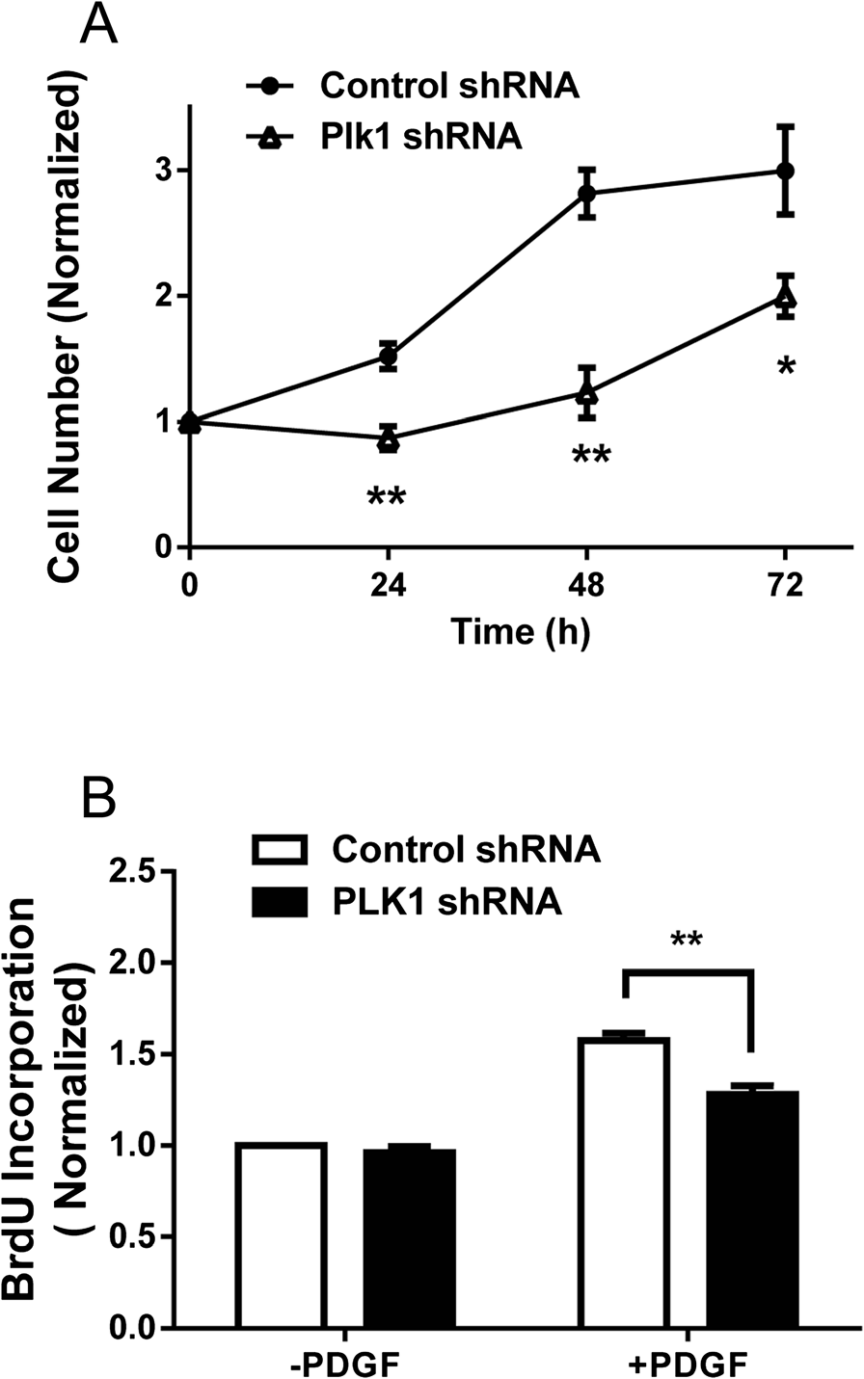
Effects of Plk1 KD on smooth muscle cell proliferation. **a** Smooth muscle cells expressing control shRNA or Plk1 shRNA were treated with 20 ng/ml PDGF for 24–72 h. The numbers of viable cells were then determined. Plk1 KD inhibits smooth muscle cell proliferation. Data are mean ± SE (n = 4, * *P* < 0.05, **P < 0.01). **b** Smooth muscle cells expressing control shRNA or Plk1 shRNA were treated with or without 20 ng/ml PDGF for 24 h. DNA synthesis in these cells was evaluated using the BrdU incorporation assay. Newly-synthesized DNA was reduced in Plk1 KD cells. Data are mean ± SE (*n* = 6, ** *P* < 0.01)

We assessed the effects of Plk1 KD on cell proliferation. Smooth muscle cells were treated with PDGF for 24–72 h. The numbers of smooth muscle cells were then evaluated. Compared to cells expressing control shRNA, the numbers of Plk1 KD cells were reduced (Fig. 4a).

We also used the BrdU incorporation assay to assess the effects of Plk1 on DNA synthesis. Basal BrdU incorporation was not different between cells expressing control shRNA and Plk1 KD cells. However, BrdU incorporated into newly synthesized DNA was lower in Plk1 KD cells than in cells expressing control shRNA (Fig. 4b).

### Plk1 silencing does not affect AKT phosphorylation in smooth muscle cells

**Fig. 5:**
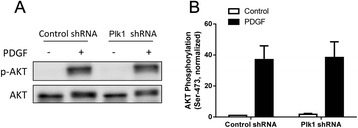
AKT phosphorylation stimulated by PDGF is not reduced in Plk1 KD smooth muscle cells. Stable Plk1 KD cells and cells expressing control shRNA were treated with 20 ng/ml PDGF for 10 min, or left untreated. **a** Representative immunoblots illustrating the effects of Plk1 KD on AKT phosphorylation. **b** Basal and the PDGF-induced AKT phosphorylation (Ser-473) is similar in Plk1 KD cells and cells producing control shRNA (*P* > 0.05). Stimulated AKT phosphorylation was normalized to the basal level of cells expressing control shRNA. Values are mean ± SE (*n* = 4)

In addition to MEK1/2 and ERK1/2 activation, exposure to growth factors also activates AKT/PKB in smooth muscle cells [9, 23]. To determine whether AKT activation is regulated by Plk1, we assessed the effects of Plk1 KD on AKT phosphorylation in cells. Stimulation with PDGF induced a significant increase in AKT phosphorylation in cells expressing control shRNA and Plk1-deficient cells (Fig. 5). The average increase in AKT phosphorylation in these cells was not significantly different 10 min after PDGF stimulation.

### The expression of mutant T210A Plk1 inhibits the PDGF-induced phosphorylation of MEK1/2 and ERK1/2, and cell proliferation

**Fig. 6:**
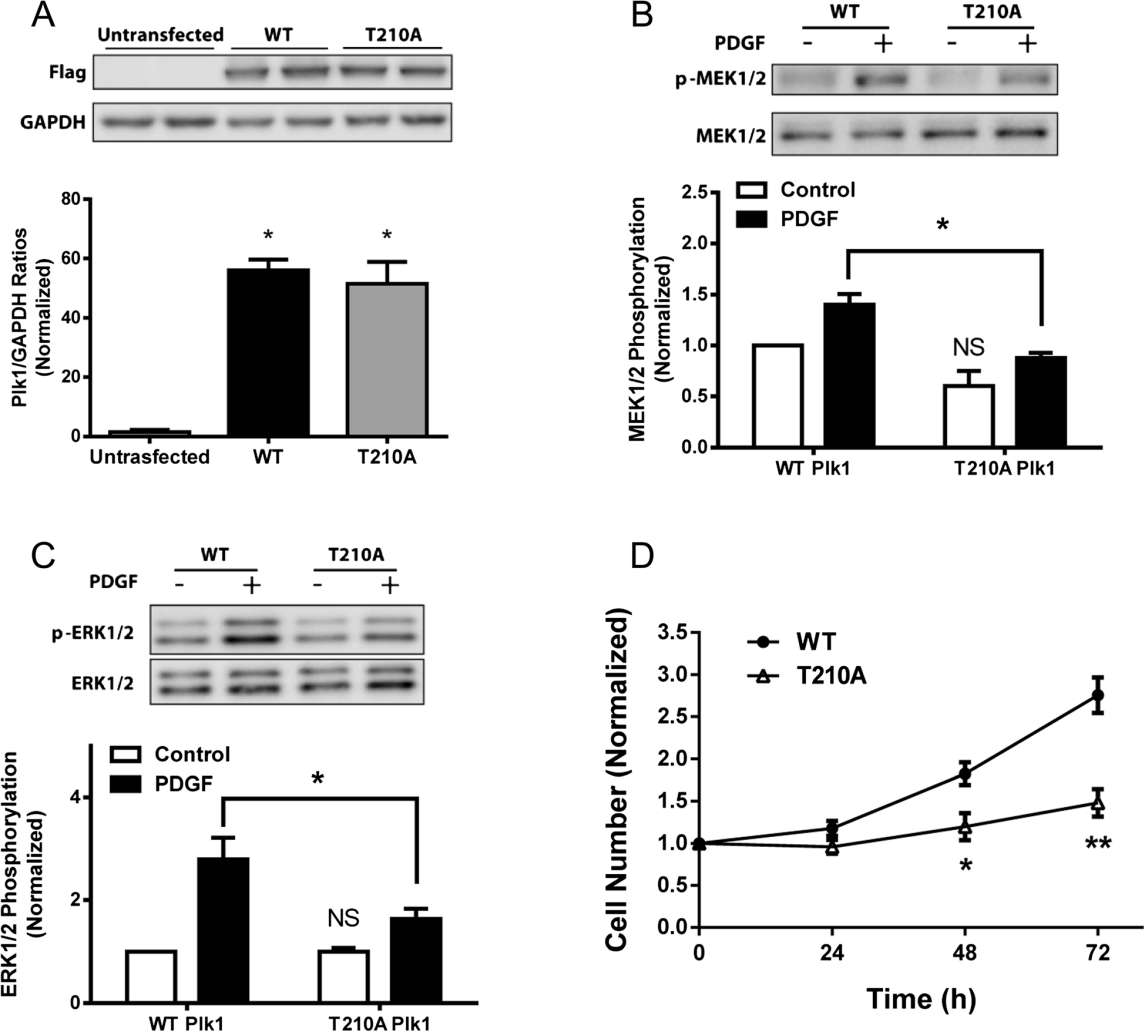
Expression of mutant T210A Plk1 inhibits the PDGF-induced phosphorylation of MEK1/2 and ERK1/2, and cell proliferation. **a** Smooth muscle cells were transfected with plasmids encoding Flag-wild type (WT) or T210A Plk1. Blots of cell extracts were detected with antibodies against Flag and GAPDH. Duplicated samples of each treatment are shown. Ratios of Plk1/GAPDH protein in cells transfected with plasmids were normalized to untransfected cells. Values are mean ± SE (*n* = 4, *P < 0.01 vs. untransfected cells). **b** and **c** Cells expressing WT or T210A Plk1 were treated with 20 ng/ml PDGF for 10 min, or they were not treated. Protein phosphorylation was then evaluated by immunoblotting. MEK1/2 phosphorylation (*n* = 5) and ERK1/2 phosphorylation (*n* = 4) induced by PDGF was significantly reduced in cells expressing T210A Plk1 compared to cells expressing WT Plk1 (*P < 0.05). Protein phosphorylation under various treatments was normalized to the levels in unstimulated cells expressing WT Plk1. Values are mean ± SE. NS, basal MEK1/2 or ERK1/2 phosphorylation in Plk1 KD was not significantly different from cells expressing control shRNA (*P* > 0.05). **d** Smooth muscle cells expressing WT or T210A Plk1 were treated with 20 ng/ml PDGF for 24–72 h. The numbers of viable cells were then determined. Data are mean ± SE (*n* = 4, *P < 0.05, **P < 0.01)

As described earlier, PDGF stimulation induces Plk1 phosphorylation at Thr-210 (Fig. 1a). To determine the role of Thr-210 phosphorylation, we assessed the effects of mutant T210A Plk1 (alanine substitution at Thr-210) on MEK1/2 and ERK1/2 in smooth muscle cells. Immunoblot analysis verified the expression of wild type (WT) or T210A Plk1 in smooth muscle cells (Fig. 6a). In cells expressing WT Plk1, PDGF stimulation induced the phosphorylation of MEK1/2 and ERK1/2. In contrast, the PDGF-induced phosphorylation of MEK1/2 and ERK1/2 was reduced in cells expressing T210A Plk1 (Fig. 6b and c).

Furthermore, we also evaluated the effects of T210A Plk1 on cell proliferation. In cells expressing WT Plk1, PDGF treatment induced cell proliferation. However, the expression of T210A Plk1 attenuated the PDGF-induced cell proliferation (Fig. 6d).

## Discussion

The protein kinase Plk1 has been implicated in various cellular functions including mitosis and cytokinesis [2, 5]. The physiological properties of Plk1 in the early stage of the growth factor response have not been investigated. In the present study, Plk1 underwent phosphorylation at Thr-210 in quiescent smooth muscle cells within minutes of PDGF stimulation. Structural analysis reveals that Plk1 is composed of a common N-terminal catalytic domain and a C-terminal regulatory domain with highly-conserved sequences named polo-box domain (PBD), and an interdomain linker [7]. PBD is involved in an autoregulatory mechanism and spatial localization [2, 7]. When inactive, PBD binds to the catalytic domain, which inhibits the access of substrates to the catalytic domain. Plk1 phosphorylation at Thr-210 may induce the dissociation of PBD from the catalytic domain, increasing kinase activity [2, 6, 7, 24]. Thus, the stimulation with the growth factor is able to activate Plk1 in smooth muscle cells.

The activation of the MAPK pathway is a key event in the early phase of cellular responses to growth factor stimulation. Our recent studies showed that the MAPK pathway in airway smooth muscle is regulated by Raf-1 [8], which is a known target of Ras [10, 11]. Thus, it is likely that the MAPK cascade is mediated by the Ras-Raf pathway in smooth muscle. Although the role of Plk1 in cell division/proliferation is well recognized, the studies on Plk1 are largely focused on the stages of mitosis and cytokinesis [2, 4, 5]. In this report, stimulation with PDGF also induced the phosphorylation of MEK1/2 and ERK1/2, which is consistent with previous results [8, 9, 25]. Furthermore, Plk1 activation preceded MEK/ERK activation in smooth muscle cells upon PDGF stimulation. More importantly, Plk1 KD attenuated the PDGF-induced phosphorylation of MEK1/2 and ERK1/2. To the best of our knowledge, this is the first evidence to suggest that Plk1 is necessary for the activation of the MAPK pathway in smooth muscle cells upon growth factor stimulation.

In response to activation with growth factors, ERK1/2 gets phosphorylated at Thr-202/Tyr-204, which is catalyzed by the dual-specificity protein kinase MEK1/2 [8, 10]. Since Plk1 is a serine/threonine protein kinase [2, 5], it is unlikely that Plk1 catalyzes ERK1/2 directly. Previous studies by others suggest that MEK1/2 is phosphorylated at Ser-218/Ser-222 and activated by Raf kinase during activation with growth factors [10, 11]. Moreover, in response to cell adhesion to fibronectin, p21-activated kinase mediates MEK1/2 phosphorylation at Ser-298, which subsequently promotes phosphorylation of Ser-218/Ser-222 and activates MEK1/2 [26]. In the present study, Plk1 KD did not affect Raf-1 phosphorylation in smooth muscle cells. Plk1 directly catalyzed MEK phosphorylation at Ser-218/Ser-222 as evidenced by the *in vitro* kinase assay. Thus, Plk1 may regulate the ERK1/2 activation by controlling MEK1/2 phosphorylation in smooth muscle cells during stimulation with growth factors.

As described earlier, the MAPK pathway is activated within minutes in response to growth factor stimulation, which phosphorylates and activates downstream targets and promotes cell proliferation eventually (takes days) [8–11]. In this report, Plk1 KD attenuated the PDGF-induced phosphorylation of MEK1/2 and ERK1/2, DNA synthesis and cell proliferation. The results support the concept that Plk1-mediated MAPK activation (within minutes) ultimately affects smooth muscle cell proliferation.

AKT phosphorylation and activation may facilitate cell survival in response to growth factor stimulation [9, 23]. Although Plk1 regulates the MAPK pathway, KD of Plk1 did not affect the PDGF-induced phosphorylation of AKT. The results suggest that Plk1 differentially regulates the activation of MEK1/2 and AKT in smooth muscle cells. AKT phosphorylation may be regulated by PI3 kinase, PTEN, the PIKK (PI3 kinase related kinase) superfamily, mTORC2 (mammalian target of rapamycin complex 2) and DNA-PK [23].

In this report, the expression of T210A Plk1 diminished the PDGF-induced phosphorylation of MEK1/2 and ERK1/2 as well as smooth muscle cell proliferation. The results suggest that phosphorylation at Thr-210 is required for the activation of MEK1/2 and ERK1/2, and cell proliferation. Phosphorylation at this residue may increase Plk1 activity, which in turn phosphorylates MEK1/2 and activates the downstream events.

ERK1/2 has been shown to control transcription factors (Elk-1, Ets-2), ribosomal S6 kinase, MNK kinase, and cPLA2, which regulate gene transcription, protein translation and other processes that facilitate cell proliferation [27, 28]. Future investigations are required to assess whether these molecules are involved in the Plk1/MAPK-regulated proliferation in smooth muscle cells. Moreover, asthmatic airway smooth muscle cells display different proliferative properties compared to non-asthmatic smooth muscle cells [1]. Since Plk1 regulates non-asthmatic airway smooth muscle cell proliferation, it would be interesting to understand whether the Plk1/MAPK pathway is altered in asthmatic airway smooth muscle cells in future studies.

How PDGF stimulation induces Plk1 phosphorylation at Thr-210 upon growth factor stimulation is currently unknown. Ste20-like kinase (SLK) is a serine/threonine protein kinase that has been implicated in spindle orientation and microtubule organization during mitosis [29–31]. *In vitro* studies suggest that SLK may catalyze Plk1 phosphorylation at Thr-210, which may facilitate G2/M transition [29, 32]. In addition, the kinase Aurora-A and its cofactor Bora are pivotal for Plk1 phosphorylation at Thr-210 during mitosis of human osteosarcoma U2OS cells [4]. Furthermore, phosphatidylinositol 3-kinase (PI3K) has been implicated in Plk1 activation during mitotic phase of Hela cells [33]. Future studies are needed to evaluate whether SLK, Aurora-A/Bora and PI3K are upstream regulator of Plk1 in smooth muscle cells in response to growth factor stimulation.

### Summary

Plk1 is a serine/threonine protein kinase that has been implicated in mitosis and cytokinesis. Our present study unveils a new and novel role that Plk1 plays in the regulation of smooth muscle cell proliferation. In response to activation of growth factors, Plk1 gets phosphorylated at Thr-210 and activated. Activated Plk1 may promote the phosphorylation and activation of MEK1/2, which subsequently activates ERK1/2 and smooth muscle cell proliferation. Thus, we propose that Plk1 controls cell proliferation at multiple cellular stages including the activation of MEK1/2, mitosis and cytokinesis.
